# Baseline serum Krebs von den Lungen-6 as a biomarker for the disease progression in idiopathic pulmonary fibrosis

**DOI:** 10.1038/s41598-022-12399-8

**Published:** 2022-05-20

**Authors:** Chiwook Chung, Jiwon Kim, Hyo Sin Cho, Ho Cheol Kim

**Affiliations:** 1 Department of Pulmonary and Critical Care Medicine, Asan Medical Center, University of Ulsan College of Medicine, 88 Olympic-ro 43-gil, Songpa-gu, Seoul, 05505 Republic of Korea; 2 University of Ulsan College of Medicine, Seoul, Republic of Korea

**Keywords:** Biomarkers, Diseases

## Abstract

Disease progression (DP) is an important parameter for the prognosis of idiopathic pulmonary fibrosis (IPF). This study aimed to evaluate the baseline serum biomarkers for predicting the DP in IPF. Seventy-four patients who were diagnosed with IPF and had their serum Krebs von den Lungen-6 (KL-6) and monocyte count, which might be associated with prognosis of IPF, checked more than twice were included. KL-6 ≥ 1000 U/mL and monocyte ≥ 600/μL were arbitrarily set as the cut-off values for DP. The DP was defined as a 10% reduction in forced vital capacity, a 15% reduction in diffusing capacity of the lung for carbon monoxide relative to the baseline, or disease-related mortality. Of the 74 patients, 18 (24.3%) were defined as having DP. The baseline KL-6 level was significantly increased in the DP group compared to the stable disease group (median, 1228.0 U/mL vs. 605.5 U/mL, *P* = 0.019). Multivariate Cox analyses demonstrated that a high KL-6 level (KL-6 ≥ 1000 U/mL; hazard ratio, 2.761 or 2.845; *P* = 0.040 or 0.045) was independently associated with DP in each model. The baseline serum KL-6 level might be a useful biomarker for DP in IPF.

## Introduction

Idiopathic pulmonary fibrosis (IPF) is a specific form of chronic, progressive, fibrosing interstitial pneumonia of unknown cause^1^. Although patients with IPF have a poor prognosis, with a median survival of 3–5 years, the clinical courses of these patients are highly variable^2,3^. Various clinical parameters are known to predict the prognosis of IPF, including older age, smoking history, lower body mass index (BMI), poorer lung function, greater disease extent, and various comorbidities^4^.

Among these parameters, physiologic parameters that can be obtained from the pulmonary function test (PFT) provide crucial information for managing patients with IPF^5^. Several studies have shown that disease progression (DP), which is defined as a decline in lung volume (forced vital capacity [FVC]) and/or a decline in gas exchange (diffusing capacity of the lung for carbon monoxide [DLco]) is associated with poor prognosis in patients with IPF^4,6^. Indeed, these physiologic parameters served as the primary end-point in previous clinical trials of anti-fibrotic agents^7,8^.

Blood biomarkers have been shown to emerging evidence in patients with IPF in clinical practice^9,10^. Several blood biomarkers, including lung-epithelium specific proteins, chemokines, growth factors, proteases, and other serological markers, have been studied widely in the fields of diagnosis, disease severity, and prognosis in patients with IPF^11–13^. Among them, Krebs von den Lungen-6 (KL-6) is used in clinical practice and elevated level of KL-6 is known to predict mortality in patients with IPF^14–17^. Recently, increased monocyte count is reported to be associated with poor outcomes in patients with IPF^18–21^. However, the role of biomarkers in predicting the DP of patients with IPF is ill-defined. Additionally, the majority of studied biomarkers are difficult to use in real world clinical practice. Therefore, this study evaluated the predictive values of serum biomarkers, including KL-6 and monocyte count, for predicting the DP in IPF.

## Results

### Baseline characteristics

Eighteen patients (24.3%) were classified as having DP according to the abovementioned criteria. The median follow-up period was 9 months. The mean age of the patients was 67.9 years, 67 patients (90.5%) were male, and 65 patients (87.8%) had smoking history. The DP group had a higher level of baseline KL-6 (median 1228.0 vs. 605.5 U/mL, *P* = 0.019) than the stable disease (SD) group (n = 56). However, there was no significant difference in baseline monocyte count between the DP and SD group (median 633.0 vs. 596.5/μL, *P* = 0.427). Additionally, there were no significant differences between the two groups in demographics, lung function, and exercise capacity (Table 1). As for anti-fibrotic medications, 64 patients (86.5%), 1 patient (1.4%), and 1 patient (1.4%) were prescribed pirfenidone, nintedanib, and both pirfenidone and nintedanib, respectively (data not shown).

**Table 1:** Comparison of baseline characteristics in the stable disease vs. disease progression group.

	Total patients (n = 74)	Stable disease (n = 56)	Disease progression (n = 18)	*P*-value
Age (years)	67.9 ± 9.0	67.3 ± 9.4	69.8 ± 7.7	0.319
Male sex	67 (90.5)	52 (92.9)	15 (83.3)	0.350
Ever-smoker	65 (87.8)	50 (89.3)	15 (83.3)	0.679
BMI (kg/m^2^)	25.2 ± 3.4	25.4 ± 3.3	24.6 ± 3.8	0.394
**Diagnosis of IPF**				> 0.999
Histopathologic	11 (14.9)	9 (16.1)	2 (11.1)	
Radiologic	63 (85.1)	47 (83.9)	16 (88.9)	
UIP	49 (66.2)	35 (62.5)	14 (77.8)	
Probable UIP	14 (18.9)	12 (21.4)	2 (11.1)	
Baseline KL-6, U/mL, median [IQR]	685.5 [394.5–1268.4]	605.5 [378.7–1008.2]	1228.0 [566.1–1945.5]	0.019
Baseline monocyte, cells/μL, median [IQR]	603.5 [494.8–729.8]	596.5 [473.8–698.8]	633.0 [515.5–910.8]	0.427
**Pulmonary function test**				
FEV_1_ (% predicted)	79.9 ± 13.8	79.9 ± 13.3	79.6 ± 15.4	0.933
FVC (% predicted)	72.0 ± 12.3	71.8 ± 11.7	72.8 ± 14.3	0.751
DLco (% predicted)	55.8 ± 18.0	56.8 ± 18.0	52.4 ± 18.0	0.361
TLC (% predicted) (n = 51)	71.8 ± 11.8	71.8 ± 11.4	71.9 ± 13.8	0.979
6MWT, distance, meter (n = 54)	428.2 ± 98.5	439.7 ± 95.5	395.4 ± 103.0	0.149
6MWT, lowest SpO_2_, % (n = 54)	90.8 ± 5.3	91.4 ± 4.7	88.9 ± 6.6	0.194

Data are presented as the mean ± standard deviation or number (%), unless otherwise indicated.

BMI: Body mass index, IPF: Idiopathic pulmonary fibrosis, UIP: Usual interstitial pneumonia, KL-6: Krebs von den Lungen-6, IQR: Interquartile range, FEV_1_: Forced expiratory volume at 1 s, FVC: Forced vital capacity, DLco: Diffusing capacity of the lung for carbon monoxide, TLC: Total lung capacity, 6MWT: 6-min walk test, SpO_2_: Saturation of percutaneous oxygen.

The baseline characteristics of the high KL-6 level (≥ 1000 U/mL) group and the low KL-6 level (< 1000 U/mL) group are shown in Table 2. The high KL-6 group had higher monocyte count (median 660.0 vs. 567.0/μL, *P* = 0.030), lower DLco (47.6 vs. 60.0%, *P* = 0.004), and lower O_2_ saturation in the 6-min walk test (6MWT) (87.6 vs. 93.0%, *P* = 0.001) than the low KL-6 group. However, there was no significant difference between the two groups in demographics and other lung function and exercise capacity.

**Table 2:** Comparison of baseline characteristics in the serum KL-6 < 1000 U/mL vs. KL-6 ≥ 1000 U/mL group.

	Total patients (n = 74)	KL-6 < 1000 U/mL (n = 49)	KL-6 ≥ 1000 U/mL (n = 25)	*P*-value
Age (years)	67.9 ± 9.0	68.5 ± 8.8	66.8 ± 9.6	0.434
Male sex	67 (90.5)	46 (93.9)	21 (84.0)	0.217
Ever-smoker	65 (87.8)	44 (89.8)	21 (84.0)	0.476
BMI (kg/m^2^)	25.2 ± 3.4	25.1 ± 3.0	25.4 ± 4.3	0.716
**Diagnosis of IPF**				> 0.999
Histopathologic	11 (14.9)	7 (14.3)	4 (16.0)	
Radiologic	63 (85.1)	42 (85.7)	21 (84.0)	
UIP	49 (66.2)	30 (61.2)	19 (76.0)	
Probable UIP	14 (18.9)	12 (24.5)	2 (8.0)	
Baseline KL-6, U/mL, median [IQR]	685.5 [394.5–1268.4]	493.1 [360.3–729.3]	1628.3 [1241.8–2473.4]	< 0.001
Baseline monocyte, cells/μL, median [IQR]	603.5 [494.8–729.8]	567.0 [446.0–696.5]	660.0 [565.5–872.0]	0.030
**Pulmonary function test**				
FEV_1_ (% predicted)	79.9 ± 13.8	81.1 ± 12.8	77.3 ± 15.5	0.261
FVC (% predicted)	72.0 ± 12.3	73.4 ± 11.8	69.4 ± 13.0	0.190
DLco (% predicted)	55.8 ± 18.0	60.0 ± 16.9	47.6 ± 17.5	0.004
TLC (% predicted) (n = 51)	71.8 ± 11.8	73.6 ± 11.4	68.7 ± 12.3	0.642
6MWT, distance, meter (n = 54)	428.2 ± 98.5	438.3 ± 101.0	413.5 ± 95.0	0.367
6MWT, lowest SpO_2_, % (n = 54)	90.8 ± 5.3	93.0 ± 3.1	87.6 ± 6.2	0.001

Data are presented as the mean ± standard deviation or number (%), unless otherwise indicated.

BMI: Body mass index, IPF: Idiopathic pulmonary fibrosis, UIP: Usual interstitial pneumonia, KL-6: Krebs von den Lungen-6, IQR: Interquartile range, FEV_1_: Forced expiratory volume at 1 s, FVC: Forced vital capacity, DLco: Diffusing capacity of the lung for carbon monoxide, TLC: Total lung capacity, 6MWT: 6-min walk test, SpO_2_: Saturation of percutaneous oxygen.

### Correlations between baseline biomarkers and lung function

**Figure 1:**
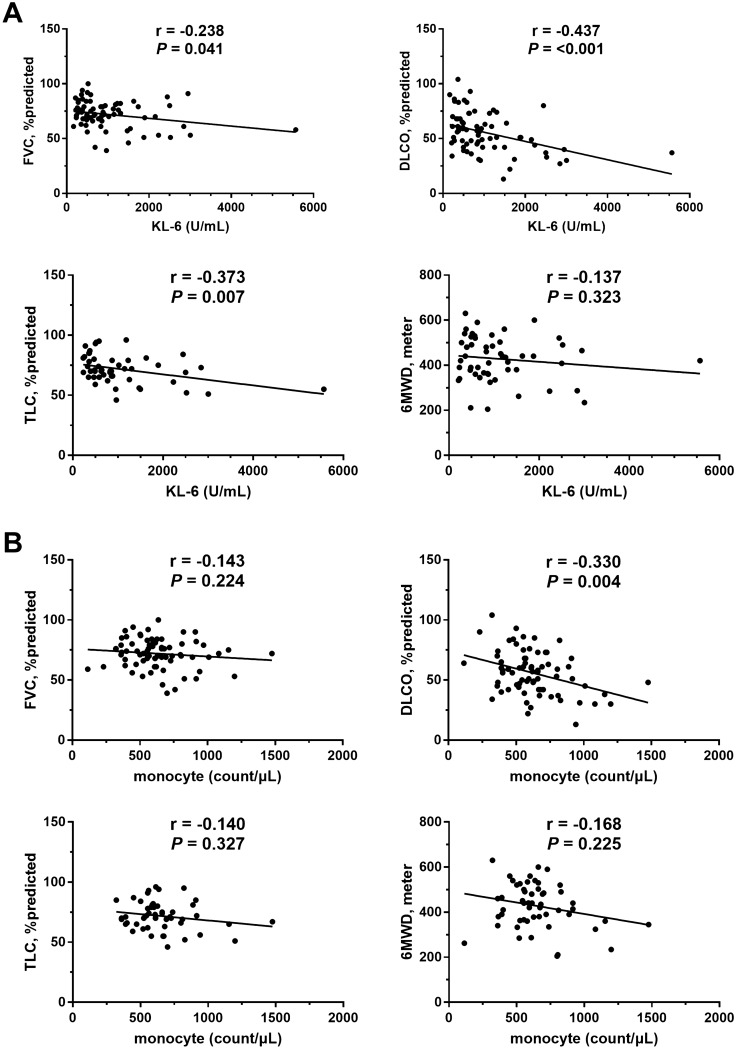
Correlation between baseline biomarkers and lung function or exercise capacity. (**A**) KL-6, (**B**) monocyte. KL-6: Krebs von den Lungen-6, FVC: Forced vital capacity, DLco: Diffusing capacity of the lung for carbon monoxide, TLC: Total lung capacity, 6MWD: 6-min walk test distance.

Baseline KL-6 levels were inversely correlated with FVC (r =  − 0.238, *P* = 0.041), DLco (r =  − 0.437, *P* < 0.001), and total lung capacity (TLC) (r =  − 0.373, *P* = 0.007). The baseline KL-6 levels were not correlated with 6MWT distance (r =  − 0.137, *P* = 0.323), while the baseline monocyte counts were inversely correlated with DLco (r =  − 0.330, *P* = 0.004), but not with FVC, TLC, and 6MWT distance (Fig. 1).

### Risk factors for disease progression

**Figure 2:**
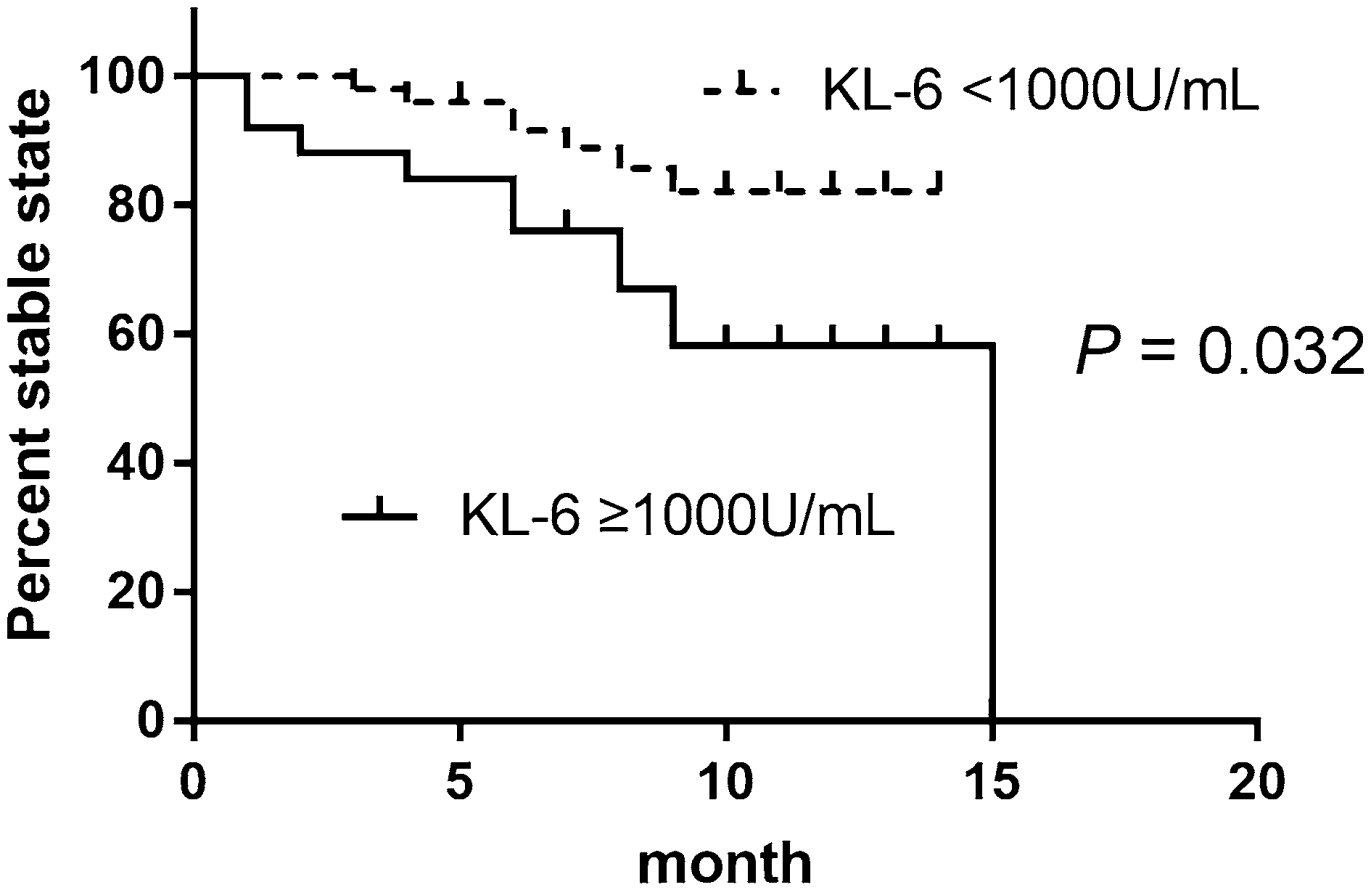
Disease progression according to the baseline KL-6 level groups. KL-6: Krebs von den Lungen-6.

Univariate analysis demonstrated that a high KL-6 level (≥ 1000 U/mL) was significantly associated with DP, while a high monocyte count (≥ 600/μL) was not. Multivariate analysis demonstrated that a high KL-6 level was independently associated with DP (hazard ratio, 2.761; 95% confidence interval, 1.048–7.273; *P* = 0.040), after adjusting for BMI. When including clinically significant variables, multivariate analysis demonstrated that a high KL-6 level was independently associated with DP (hazard ratio, 2.845; 95% confidence interval, 1.023–7.912; *P* = 0.045), after adjusting for age, sex, BMI, forced expiratory volume at 1 s (FEV1), FVC, and DL_CO_ (Table 3). The Kaplan–Meier curve demonstrated that the high KL-6 group showed a higher risk of DP than the low KL-6 group (*P* = 0.032, Fig. 2). However, the high monocyte count group showed no significant differences from the low monocyte count group (*P* = 0.440).

**Table 3:** Risk factors for disease progression in patients with IPF by Cox proportional hazards model.

Parameter	Hazard ratio	95% confidence interval	*P*-value
**Univariate analysis**			
Age (years)	1.022	0.966–1.082	0.446
Male sex	0.493	0.141–1.718	0.267
Ever-smoker	0.656	0.188–2.288	0.509
BMI (kg/m^2^)	0.904	0.778–1.051	0.189
Baseline KL-6 ≥ 1000 U/mL	2.718	1.033–7.154	0.043
Baseline monocyte ≥ 600/μL	1.451	0.552–3.812	0.450
FEV_1_ (% predicted)	1.006	0.969–1.044	0.768
FVC (% predicted)	1.020	0.979–1.063	0.348
DLco (% predicted)	0.988	0.960–1.015	0.378
TLC (% predicted)	1.006	0.954–1.060	0.832
6MWT, distance, meter	0.997	0.992–1.003	0.304
6MWT, lowest SpO_2_, %	0.959	0.875–1.052	0.376
**Multivariate analysis 1**			
BMI (kg/m^2^)	0.910	0.795–1.043	0.177
Baseline KL-6 ≥ 1000 U/mL	2.761	1.048–7.273	0.040
**Multivariate analysis 2**			
Baseline KL-6 ≥ 1000 U/mL	2.845	1.023–7.912	0.045
FVC (% predicted)	1.050	0.993–1.110	0.085

IPF: Idiopathic pulmonary fibrosis, BMI: Body mass index, KL-6: Krebs von den Lungen-6, FEV_1_: Forced expiratory volume at 1 s, FVC: Forced vital capacity, DLco: Diffusing capacity of the lung for carbon monoxide, TLC: Total lung capacity, 6MWT: 6 min walk test, SpO,: Saturation of percutaneous oxygen.

Multivariate analysis 1 included variables with *P* < 0.2 on univariate analysis.

Multivariate analysis 2 included variables with *P* < 0.2 on univariate analysis and variables with clinical significance, including age, sex, FEV1, FVC, and DL_CO_.

**Figure 3:**
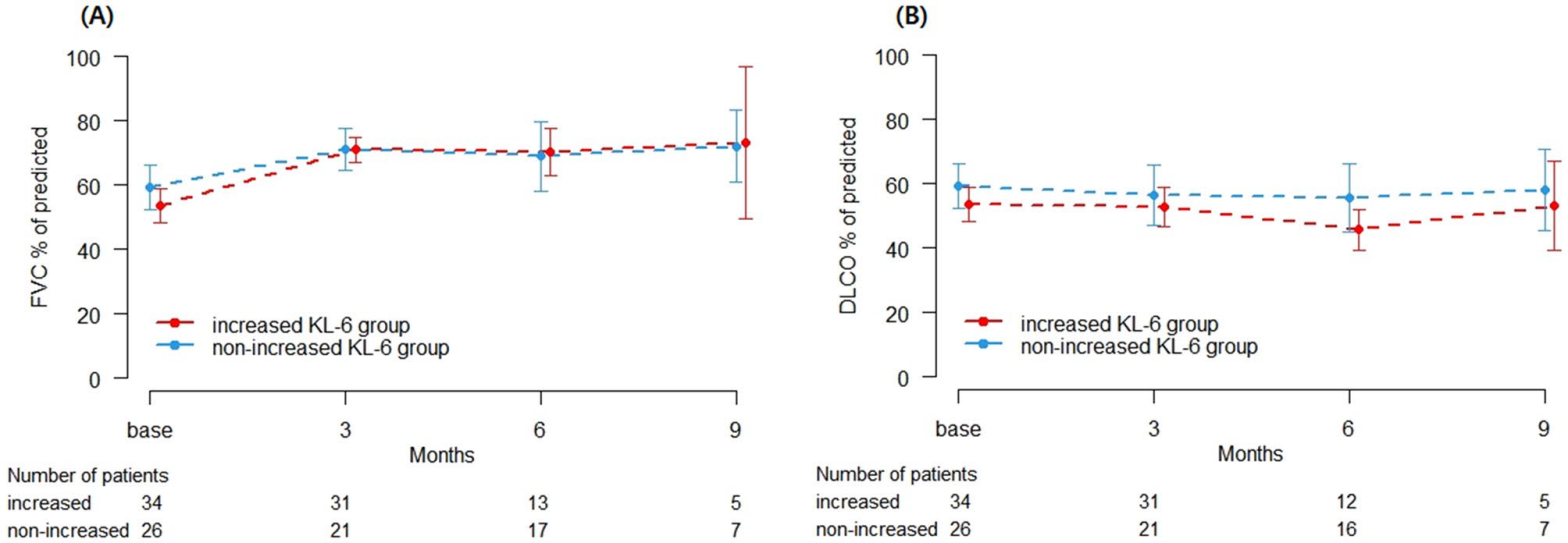
Serial lung function change according to the KL-6 level by serial change groups. (**A**) FVC, (**B**) DLco. KL-6: Krebs von den Lungen-6, FVC: Forced vital capacity, DLco: Diffusing capacity of the lung for carbon monoxide.

We also compared lung function between the increased KL-6 group and the non-increased KL-6 group to determine whether the interval change of KL-6 level is associated with DP. The increased KL-6 group (n = 34) and the non-increased KL-6 group (n = 26) showed no significant differences in the proportion of DP (*P* = 0.817, Table 4). Moreover, the serially measured lung functions at the 3rd, 6th, and 9th months from baseline were not significantly different between the groups (FVC; *P* = 0.590, DLco; *P* = 0.964, Fig. 3).

**Table 4:** Comparison of disease progression in the increased KL-6 vs. non-increased KL-6 group.

	Total patients (n = 60)	Increased KL-6 (n = 34)	Non-increased KL-6 (n = 26)	*P-* value
Age (years)	68.3 ± 8.8	69.4 ± 8.2	66.8 ± 9.5	0.255
Male sex	53 (88.3)	30 (88.2)	23 (88.5)	> 0.999
Ever-smoker	51 (85.0)	28 (82.4)	23 (88.5)	0.719
**Disease status**				0.817
Disease progression	13 (21.7)	7 (20.6)	6 (23.1)	
Stable disease	47 (78.3)	27 (79.4)	20 (76.9)	

Data are presented as the mean ± standard deviation or number (%), unless otherwise indicated.

KL-6: Krebs von den Lungen-6.

## Discussion

In the current study, the baseline serum KL-6 levels increased in the DP group compared to the SD group in patients with IPF. The baseline serum KL-6 level was independently associated with DP in multivariate analysis. These findings suggest that the baseline serum KL-6 level might be a useful predictive marker for the DP in IPF.

Previous studies have reported that several factors are associated with mortality in IPF, including age, sex, smoking status, dyspnea, BMI, pulmonary hypertension, PFT, 6MWT, and serum and bronchoalveolar lavage fluid biomarkers^4,22^. Among these factors, declined lung function is one of the best surrogate markers for disease-related mortality in IPF^23^. In particular, FVC and DL_CO_ are the only physiologic variables in the IPF staging system GAP index^24^ and they are also used for defining the DP in IPF^22^. Thus, FVC and DL_CO_ are considered to be good indicators of lung function and prognosis in IPF. As a result, reducing the decline of FVC is an important goal in IPF treatment and is regarded as the primary outcome of studies on anti-fibrotic drugs^7,8^. However, few studies have focused on the predictors of DP in IPF, especially using biomarkers.

Biomarkers might have advantages in reproducibility because they are less influenced by patients’ general conditions or examiners’ variations. Many potential biomarkers have been studied, including KL-6, SP-A, SP-D, MMPs, and CCLs^9–13^. Among them, KL-6 is well established and used in clinical practice^14–17^. KL-6 is a mucin-like glycoprotein and is strongly expressed on the surface of type II pneumocytes, which are regenerated over the alveolar basement membrane after the death of type I pneumocytes during the lung injury in interstitial lung disease^25^. Patients with IPF with an elevated level of KL-6 (KL-6 > 1000 U/mL) are known to have increased mortality compared to patients with a KL-6 level within the normal range^17,26^. In this study, baseline KL-6 ≥ 1000 U/mL was significantly associated with the DP in IPF, suggesting that KL-6 might act as a surrogate marker for DP in IPF.

There are conflicting studies regarding the values of serial changes in serum KL-6 levels for the prognosis in IPF. Wakamatsu et al. reported that patients with increased serum KL-6 levels during follow-up had significant decline in FVC compared to those with no KL-6 increase^15^. d’Alessandro et al. also showed that patients with persistently elevated KL-6 had decreased FVC, whereas patients with decreased KL-6 had preserved FVC^27^. However, Ishii et al. suggested that a reduction in serum KL-6 level with untreated IPF does not indicate stable IPF status, but DP in IPF with a natural decline in serum KL-6 level^28^. It is possible that the serum KL-6 level reflects the extent of damaged alveolar epithelium^25^ rather than the degree of disease activity. In this study, the increased KL-6 group showed no significant difference in DP and lung function compared to the non-increased KL-6 group. Therefore, our results suggest that patients with high level of baseline serum KL-6 should be considered to have the higher possibility of DP regardless of the follow-up serum KL-6 level. However, it should be considered that patients with severely deteriorated lung function, including deceased, could not perform follow-up lung function test, and therefore may not have been included in the analysis. Further studies will be needed for the serial measurement of KL-6 level.

Monocytes are the largest type of white blood cell. Monocytes can migrate from blood vessels into tissues, where they differentiate into macrophages and dendritic cells^29^. Monocyte-derived alveolar macrophages can contribute to lung fibrosis, whereas tissue-resident macrophages do not contribute to fibrosis^30^. An increased monocyte count is associated with poor outcomes in IPF and other fibrotic diseases, including systemic sclerosis, hypertrophic cardiomyopathy, or myelofibrosis^18^. However, the optimal cut-off value of monocyte count for the prognosis of IPF is still debated. Scott et al. and Teoh et al. reported that a monocyte count ≥ 950/μL was associated with mortality in IPF^18,19^. Karampitsakos et al. suggested that a monocyte count ≥ 600/μL was related to lower lung function and higher all-cause mortality^20^. Kreuter et al. showed that a monocyte count ≥ 600/μL was associated with increased risks of IPF progression, all-cause hospitalization, and all-cause mortality^21^. Considering the results of previous studies, and the fact that only six patients (8%) had a monocyte count ≥ 950/μL in our study, we arbitrarily set a monocyte count ≥ 600/μL as the cut-off value. However, the high monocyte count was not associated with the DP in IPF in our data, possibly because our sample size was small to validate it or because of the influence of other situations, including other co-incidental infections.

This study has some limitations. First, this was a single-center, retrospective, nonrandomized study which might induce a selection bias. Second, the monocyte count was calculated by white blood cell differential on complete blood count (CBC) rather than flow cytometry, so the calculated monocyte count may differ from the real value. However, previous studies checked the monocyte count calculated by white blood cell differential on CBC and showed the associations between the monocyte count and the prognosis in IPF^20,21^. Third, the follow-up periods were relatively short, with a median follow-up of 9 months. However, approximately one-fourth of patients were classified as having DP, which was sufficient compared to previous studies^14,17^. Fourth, subgroup analysis according to the anti-fibrotic medication profile could not be performed due to our small sample. Despite these limitations, our study revealed the clinical role of KL-6 in predicting DP of patients with IPF.

This real-world population analysis suggests that elevated baseline serum KL-6 level might predict the DP in IPF and it could be a useful biomarker in clinical practice.

## Methods

### Study population

Seventy-four patients who were diagnosed with IPF and had their KL-6 and monocyte count checked more than twice between January 2020 and June 2021 at Asan Medical Center, Seoul, Republic of Korea were included in this study. All patients underwent thorough systemic history taking. Patients with definite connective tissue disease or an exposure history in relation to the possible causes of interstitial lung disease were excluded. IPF diagnosis was performed by a multidisciplinary approach, using the diagnostic criteria set by the American Thoracic Society (ATS)/European Respiratory Society (ERS)/Japanese Respiratory Society/Latin American Thoracic Association in 2018^1^. All available baseline clinical parameters were obtained within 3 months of the initial blood sampling time. The study protocol was approved by the Institutional Review Board of Asan Medical Center (approval number: 2021–0787). The requirement for informed consent was waived because of the retrospective study design. The present study complied with the Declaration of Helsinki and all methods were performed in accordance with the relevant guidelines and regulations.

### Serum biomarkers

As biomarkers, serum KL-6 and monocyte count were evaluated in this study. The serum KL-6 concentration was measured using a Nanopia KL-6 assay (SEKISUI MEDICAL, Tokyo) using a latex-enhanced immunoturbidimetric assay method. The monocyte count was calculated using white blood cell differential on CBC. Serum KL-6 ≥ 1000 U/mL is well known to predict poor prognosis in IPF, and, as such, was set as the cut-off value in this study^15,17,26^. However, the optimal cut-off value of monocytes has not yet been validated. Therefore, we arbitrarily set monocytes ≥ 600/μL as a cut-off value based on recent studies^20,21^. Moreover, to determine whether the interval change in KL-6 level is related to DP, we divided patients into two groups according to the KL-6 level at the 3rd month from the baseline. Patients who had increased KL-6 levels at the 3rd month compared to baseline level were classified as the increased KL-6 group, and other patients were classified as the non-increased KL-6 group.

### Disease progression

Spirometry, DLco, and TLC were measured according to the ERS/ATS recommendations^31,32^. The 6MWT was performed according to the ERS/ATS guidelines^33^. Baseline was defined as the time when the patients were diagnosed as IPF in our center. Patients visited outpatient clinic approximately every three months and KL-6, CBC, PFT, and 6MWT were performed at every visit as possible. Data collection closing date was June 31, 2021 and at this point, the last clinical data were compared to those of baseline. DP was defined as a 10% reduction in FVC and/or a 15% reduction in DL_CO_ relative to baseline, or disease-related mortality^22^. If the patient did not meet the DP criteria, the patient was considered to have SD.

### Statistical analysis

Continuous variables are presented as means (standard deviation) or median (interquartile range), and categorical variables are expressed as numbers (percentages). Student’s *t*-test or Mann–Whitney test was used to compare continuous variables, and the χ^2^ test or Fisher’s exact test was used to compare categorical variables. Correlation analyses using Spearman’s rank correlation coefficients were performed to evaluate the relationship between biomarker levels and lung function. Cox proportional hazard analysis was used to identify risk factors for DP. Variables with *P*-values < 0.2 on univariate analysis and clinically significant variables were entered into the multivariate models. The period from the time of initial blood sampling to the time of DP was evaluated using Kaplan–Meier survival analysis and the log-rank test. All *P*-values were two-tailed, with statistical significance set at *P* < 0.05. All statistical analyses were performed using SPSS software (version 26.0; Statistical Package for the Social Sciences, IBM Corporation, Armonk, NY, USA).

### Ethics declarations

The study protocol was approved by the Institutional Review Board of Asan Medical Center (approval number 2021–0787). The requirement for informed consent was waived because of the retrospective study design. The present study complied with the Declaration of Helsinki and all methods were performed in accordance with the relevant guidelines and regulations.
